# Risk prediction model for waitlist mortality in patients with left ventricular assist devices

**DOI:** 10.1016/j.jhlto.2025.100337

**Published:** 2025-07-19

**Authors:** Anjan Tibrewala, Duc Thinh Pham, Mo Hu, Lucia C. Petito, Jonathan D. Rich, Finn Gustafsson, Theo M.M.H. de By, Kevin Veen, Donald M. Lloyd-Jones, Sanjiv J. Shah

**Affiliations:** a Division of Cardiology, Department of Medicine, Northwestern University Feinberg School of Medicine, Chicago, IL; b Bluhm Cardiovascular Institute, Northwestern Medicine, Chicago, IL; c Division of Cardiac Surgery, Department of Surgery, Northwestern University Feinberg School of Medicine, Chicago, IL; d Department of Preventive Medicine, Northwestern University Feinberg School of Medicine, Chicago, IL; e Department of Cardiology, Rigshospitalet and Department of Clinical Medicine, University of Copenhagen, Copenhagen, Denmark; f EUROMACS Registry, EACTS House, Windsor, United Kingdom; g Department of Cardio-Thoracic Surgery, Erasmus University Medical Center, Rotterdam, Netherlands; h Division of Cardiology, Department of Medicine, Boston University School of Medicine, Boston, MA

**Keywords:** Heart failure, Machine learning, Mortality

## Abstract

**Background:**

Left ventricular assist devices (LVAD) are a bridge to heart transplantation (HT). Given limited donor organs, assessment of risk of waitlist mortality is important for waitlist prioritization for HT. We sought to derive and validate a risk prediction model for waitlist mortality in LVAD patients.

**Methods:**

Adult patients with a continuous-flow, centrifugal, durable LVAD listed or likely to be listed for HT in the Interagency Registry for Mechanically Assisted Circulatory Support (INTERMACS) were included. The outcome was time to all-cause mortality within 2 years from implant. We considered 41 candidate predictors at 3 months post-implant. Univariate Fine-Gray models and 4 logistic regression techniques (logistic, LASSO, random forest, gradient boosting) were used to select variables for a final survival model using the Fine-Gray method. The model was validated in INTERMACS and in an independent cohort (European Registry for Patients with Mechanical Circulatory Support [EUROMACS]). Model discrimination and calibration were evaluated.

**Results:**

The INTERMACS cohort included 2364 patients with 268 (11%) deaths. A risk prediction model for waitlist mortality at 2 years was derived with area-under-the-curve (AUC) of 0.72 (95% CI 0.67–0.77). The EUROMACS cohort included 577 patients with 70 (12%) deaths. The model AUC was 0.62 (95% CI 0.55–0.70). The model predicted waitlist mortality when divided into low-, medium-, or high-risk groups in the INTERMACS (p<0.001) and EUROMACS (p=0.0099) cohorts.

**Conclusions:**

We derived and validated a risk prediction model for waitlist mortality in LVAD patients using 2 independent cohorts. Our risk assessment model can inform HT prioritization in LVAD patents.

## Graphical abstract

**Figure:**
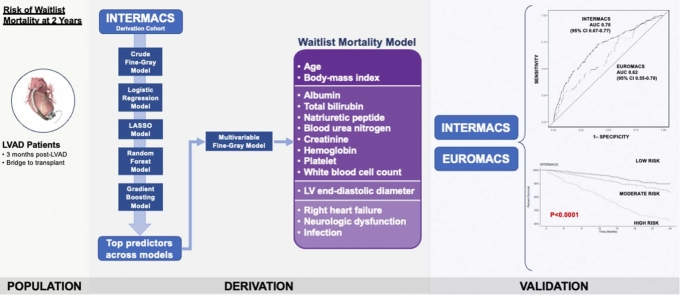

## Introduction

Advanced heart failure (HF) is a highly morbid and deadly condition.^1^ Durable left ventricular assist devices (LVAD) have become standard-of-care for this condition, used as a bridge to heart transplant (HT) or destination therapy.^2^, ^3^ Nonetheless, heart transplant remains a scarce resource. Of the LVAD patients being bridged to HT between 2018–22 in the United States, only about 25–40% received a transplant within 2 years of implantation.^4^, ^5^, ^6^ In Europe and Asia, only 9% of HeartMate 3™ LVAD patients had received HT within 2 years.^7^

Furthermore, LVAD patients are at risk of significant morbidity and mortality. Approximately 9–14% of LVAD patients listed for HT in the United States between 2018–22 died within 2 years of being waitlisted.^4^, ^5^, ^6^, ^8^ Similarly, 25% of LVAD patients died within 2 years of implant while awaiting HT in a European and Asian cohort.^7^ Many of these patients could have potentially had a more favorable outcome had a donor organ become available. Currently, LVAD patients are often uniformly prioritized on the HT waiting list largely on the basis of device-related adverse events.^9^ However, LVAD patients have unique clinical profiles to be considered including specific comorbidities, abnormal laboratory values, and hemodynamic derangements in addition to exposure to adverse events that are associated with clinical outcomes.^10^, ^11^, ^12^, ^13^ Furthermore, existing data suggests that the current HT allocation scheme inadequately discriminates risk of waitlist mortality amongst LVAD patients.^14^

Therefore, we sought to derive and validate a comprehensive risk assessment model for waitlist mortality in LVAD patients awaiting HT.

## Methods

### Study population

We utilized the Interagency Registry for Mechanically Assisted Circulatory Support (INTERMACS) as a derivation and validation cohort for a risk assessment model for waitlist mortality. INTERMACS is a multicenter registry with patient-level data on over 22,000 LVAD implants at over 170 sites in the United States.^15^ The European Registry for Patients with Mechanical Circulatory Support (EUROMACS) was used as an external validation cohort using a population of LVAD patients. EUROMACS is a multicenter registry of patient-level data on over 8000 LVAD patients at 54 sites in Europe.^16^ The study qualified for exempt status by the Institutional Review Board of Northwestern University. The study followed processes outlined by the Transparent Reporting of a Multivariable Model for Individual Prognosis or Diagnosis (TRIPOD) Statement.^17^

We identified adult patients (age ≥18 years) that underwent implantation of a durable LVAD between April 2008 and December 2017 in both cohorts. We included follow-up data until December 2019 based on data available to the investigators for this analysis. We included patients implanted with a continuous, centrifugal-flow LVAD that remained alive on LVAD support at 3 months following implantation. Specific LVAD brand (e.g. HeartMate 3™ or Heartware HVAD™) was not available in the registry. Furthermore, we included patients that were listed for heart transplant or “likely to be eligible” for heart transplant listing as defined in INTERMACS per the discretion of the implanting center. We excluded LVAD implants in patients with a pre-existing durable LVAD (i.e. pump exchange). In addition, we excluded patients on biventricular ventricular assist device (BiVAD) support or with a history of heart transplant.

### Outcome ascertainment

The primary outcome was time to all-cause mortality within 2 years of LVAD implantation, conditional on patients being alive on LVAD support at 3 months following implant. Competing events that were considered included HT or LVAD explant within 2 years of LVAD implant. All patients were censored at 2 years since most LVAD patients listed for HT that have a clinical event (e.g. HT or death) do so within 2 years of LVAD implantation.^6^, ^8^, ^18^

### Candidate predictor variables

We evaluated several variables from INTERMACS and EUROMACS including patient demographics, co-morbidities, laboratory values, imaging and hemodynamic measurements, and occurrence of adverse events. Of the numerous variables available in the registries, we selected 41 for the analysis based on clinical relevance in relation to the primary outcome. These variables are listed in Table 1.

**Table 1:** Baseline Characteristics of INTERMACS and EUROMACS Cohorts.*

Characteristic	INTERMACS (n=2364)	EURORMACS (n=577)	P-value
Demographics			
Age	56.0 [46.0, 63.0]	55.0 [47.0, 60.0]	0.005
Male	1793 (76.0)	494 (85.6)	<0.001
BMI	27.4 [23.7, 31.3]	25.8 [23.0, 29.0]	<0.001
Race			<0.001
White	1456 (61.6)	502 (89.8)	
Black	584 (24.7)	1 (0.2)	
American Indian/Alaska Native	22 (0.9)	0 (0.0)	
Asian	52 (2.2)	35 (6.3)	
Hispanic/Latino	176 (7.4)	0 (0.0)	
Unknown	74 (3.1)	21 (3.8)	
Comorbidities			
Primary heart failure diagnosis			<0.001
Ischemic	800 (34.1)	288 (49.9)	
Nonischemic	1458 (62.1)	276 (47.8)	
Congenital	21 (0.9)	0 (0.0)	
Hypertrophic	22 (0.9)	5 (0.9)	
Restrictive	25 (1.1)	0 (0.0)	
Valvular	21 (0.9)	5 (0.9)	
Diabetes mellitus	152 (6.4)	56 (9.9)	<0.001
Pre-LVAD stroke	88 (3.7)	18 (3.1)	0.567
Peripheral vascular disease	53 (2.2)	22 (5.6)	<0.001
Dialysis	112 (4.7)	58 (10.1)	<0.001
Cardiac arrhythmia	502 (21.2)	66 (11.4)	<0.001
Ventricular arrhythmia	217 (9.2)	28 (4.9)	0.001
Supraventricular arrhythmia	267 (11.3)	27 (4.7)	<0.001
Laboratory data			
Blood type			<0.001
O	1204 (51.2)	184 (31.9)	
A	793 (33.7)	283 (49.0)	
B	284 (12.1)	76 (13.2)	
AB	72 (3.1)	34 (5.9)	
White blood cell (x10^3^/uL)	7.1 [5.8, 8.7]	7.6 [6.5, 9.4]	<0.001
Hemoglobin (g/dL)	11.2 [10.0, 12.6]	11.6 [10.6, 12.8]	<0.001
Platelet (x10^3^/uL)	246.0 [200.0, 296.0]	255.0 [217.0, 304.0]	0.001
Lactate dehydrogenase (U/L)	241.0 [206.0, 290.0]	249.0 [211.0, 305.0]	0.004
International normalized ratio	2.3 [1.9, 2.7]	2.4 [2.1, 2.8]	<0.001
Sodium (mEq/L)	138.0 [136.0, 140.0]	138.0 [136.0, 140.0]	0.444
Blood urea nitrogen (mg/dL)	19.0 [14.0, 26.0]	41.0 [27.1, 70.1]	<0.001
Creatinine (mg/dL)	1.1 [0.9, 1.4]	1.1 [0.9, 1.6]	0.531
C-reactive protein (mg/L)	6.7 [2.0, 24.3]	1.0 [0.4, 2.6]	<0.001
Albumin (g/dL)	3.8 [3.5, 4.2]	4.0 [3.7, 4.3]	<0.001
Total bilirubin (mg/dL)	0.6 [0.4, 0.9]	0.6 [0.4, 0.9]	0.255
BNP (pg/mL)	396.0 [216.0, 819.0]	523.5 [239.5, 915.0]	0.146
NTproBNP (pg/mL)	2174.0 [1139.0, 4235.0]	2066.0 [1236.0, 3674.0]	0.694
Echocardiographic parameters			
LV end-diastolic diameter (cm)	6.3 [5.5, 7.1]	6.3 [5.1, 7.1]	0.213
RV dysfunction			<0.001
None	128 (5.5)	39 (15.2)	
Mild	287 (12.3)	60 (23.4)	
Moderate	352 (15.1)	105 (41.0)	
Severe	262 (11.2)	36 (14.1)	
Unknown/not measured	1335 (56.4)	326 (56.5)	
Mitral regurgitation			<0.001
None	252 (10.7)	113 (19.6)	
Mild	620 (26.2)	217 (37.6)	
Moderate	172 (7.3)	40 (6.9)	
Severe	53 (2.2)	0 (0.0)	
Unknown/not measured	1267 (53.6)	207 (35.9)	
Tricuspid regurgitation			<0.001
None	164 (6.9)	95 (16.5)	
Mild	671 (28.4)	222 (38.5)	
Moderate	197 (8.3)	68 (11.8)	
Severe	64 (2.7)	0 (0.0)	
Unknown/not measured	1268 (53.6)	192 (33.3)	
Aortic regurgitation			<0.001
None	540 (22.8)	212 (36.7)	
Mild	480 (20.3)	155 (26.9)	
Moderate	42 (1.8)	3 (0.5)	
Severe	9 (0.4)	0 (0.0)	
Unknown/not measured	1293 (54.7)	207 (35.9)	
Invasive hemodynamics			
Central venous pressure (mmHg)	11.0 [7.0, 15.0]	7.0 [6.5, 15.5]	0.456
Mean PA pressure (mmHg)	28.7 [22.7, 35.7]	23.7 [20.5, 34.5]	0.36
PCWP (mmHg)	21.0 [15.0, 28.0]	11.5 [10.5, 23.5]	0.063
Cardiac index (L/min/m^2^)	2.3 [1.9, 2.7]	NA	
LVAD-related adverse events**			
Right heart failure	482 (20.4)	25 (4.3)	<0.001
Device malfunction	107 (4.5)	9 (1.6)	0.002
Pump thrombosis	18 (0.8)	4 (0.7)	1.00
Infection - all sources	549 (23.2)	132 (22.9)	0.903
Infection - pump/driveline	95 (4.0)	61 (10.6)	<0.001
Neurologic dysfunction - any type	202 (8.5)	36 (6.2)	0.083
Neurologic dysfunction - stroke	116 (4.9)	16 (2.8)	0.035
Major bleeding event	386 (16.3)	48 (8.3)	<0.001

*Laboratory, echocardiographic, and hemodynamic variables were assessed at approximately 3 months following LVAD implant.

**LVAD-related adverse events occurred between implant and 3-month follow-up.

Numbers represented are n (%) or median [interquartile range].

BMI – body-mass index; INR – international normalized ratio; LV – left ventricle; LVAD – left ventricular assist device; NA – not available

Variables were measured at specific time points in INTERMACS and EUROMACS based on pre-defined forms (e.g. 3-month follow-up form in INTERMACS) and timestamps. Patient demographics and co-morbidities were assessed on patient registration forms (i.e. at time of implant). When available, laboratory, echocardiographic, and hemodynamic criteria were obtained at approximately 3-months post-implant. LVAD-related adverse events and arrhythmias were recorded if occurring anytime between device implant and 3-month follow-up.

Variable definitions were provided by the individual registries (Supplemental Appendix).^15^, ^16^ We used a creatinine value of 6 mg/dL for patients with creatinine ≥ 6 mg/dL or receiving dialysis treatments. Similarly, we used a blood urea nitrogen (BUN) value of 120 mg/dL for patients with BUN ≥ 120 mg/dL or receiving dialysis treatments. A similar methodology has been validated in the Model for End-Stage Liver Disease (MELD) score used to prioritize patients on the liver transplant waiting list.^19^, ^20^, ^21^ B-type natriuretic peptide (BNP) and N-terminal pro-B-type natriuretic peptide (NT-proBNP) values were combined into a standardized natriuretic peptide (NP) variable based on standardized mean and standard deviation values (i.e., Z-score approach).

### Statistical analysis

We first summarized and compared baseline characteristics in the derivation (INTERMACS) and validation (EUROMACS) cohorts. For continuous variables, we reported the number of available observations, median values, and interquartile ranges. For categorical variables, we reported the number of available observations, counts, and frequencies. Baseline characteristics were compared across the 2 cohorts using t-tests (or Wilcoxon rank-sum tests, when appropriate) for continuous variables and chi-squared tests (or Fisher exact tests, when appropriate) for categorical variables.

For derivation of the LVAD risk prediction model, we used the INTERMACS cohort. For data missingness, candidate predictors with data missing in ≥25% of patients were excluded from the analysis (k=8). Other missing values were imputed using chained equations.^22^ Cubic spline analysis was used to assess for non-linearity of continuous variables. Given that non-linearity was not observed in any variables, further categorization of continuous variables was not necessary. All continuous variables were standardized to mean 0 and standard deviation 1 to facilitate comparability between variables.

We used a data-science driven approach to variable selection. First, we created a dataset where individuals who died during the follow-up period were oversampled such that the event to non-event ratio was 1:1. We evaluated the univariate association between each variable and the time to death using a Fine-Gray model accounting for competing risks of transplant and explant.^23^ Then, we fit 4 multivariable model with death as the dependent variable and all candidate predictors as the independent variables: logistic regression, logistic LASSO regression (penalty selected by cross-validation), a random forest (RF) classifier, and gradient boosting machine (GBM). For each of the 5 techniques, co-variates were ranked by p-value (Fine-Gray model, logistic regression) or strength of association with the outcome (LASSO, RF, GBM). Candidate predictors that were in the top 10 using at least 2 of the 5 methods were included in the multivariable model (k=14). Once candidate predictors were selected, one final multivariable Fine-Gray model accounting for completing risks of transplant and explant was derived to predict the risk of mortality within 2 years of LVAD implantation.

The risk prediction model was validated using the INTERMACS cohort and an independent cohort of patients from the EUROMACS registry. External validation in the EUROMACS cohort was performed after the risk model was derived in the INTERMACS cohort to prevent bias in model development. Model discrimination was assessed using the area under the time-dependent receiver-operating characteristic (AUC) at 2 years using bootstrap cross-validation. Model calibration was assessed by categorizing the derivation and validation cohorts into quintiles. The average predicted and observed survival for each quintile were evaluated at 2 years in the two cohorts using a Pearson correlation coefficient. We classified patients as low, medium, or high risk using a Kaplan-Meier analysis to compare survival at 2 years. Risk thresholds were created in the derivation cohort by consolidating the quintiles of risk into 3 categories for ease of interpretation and clinical similarity. These thresholds were then applied to the validation cohort.

All statistical analyses were performed using R Version ≥3.5.0 (R Foundation for Statistical Computing, Vienna, Austria). A 5% type-I error rate was used to evaluate statistical significance.

## Results

### Study cohort

**Figure 1:**
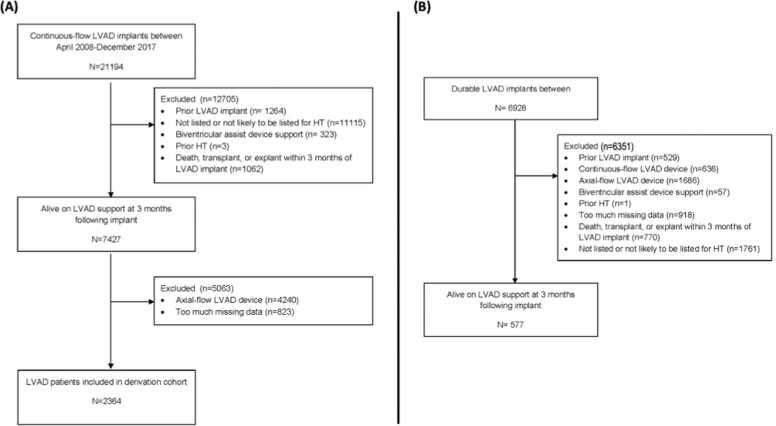
CONSORT diagrams of the (A) INTERMACS cohort and the (B) EUROMACS cohort. **Legend:** LVAD = left ventricular assist device. HT = heart transplant.

A CONSORT diagram of derivation cohort from INTERMACS is shown (Figure 1A). There were 2364 patients used for analysis in the derivation cohort. A CONSORT diagram of the validation cohort from EUROMACS is shown (Figure 1B). There were 577 patients used for analysis in the validation cohort.

### Cohort characteristics and two-year survival

The clinical characteristics of patients in the derivation (INTERMACS) and validation (EUROMACS) cohorts are shown in Table 1. Notably, patients in the INTERMACS cohort had significantly higher rates of arrhythmias, right heart failure, device malfunction, LVAD-related stroke, and major bleeding events. Patients in the EUROMACS cohort had significantly higher rates of dialysis and pump/driveline infection.

### Model derivation

In the derivation cohort, there were 268 (11%) deaths, 1280 (54%) transplants, and 148 (6%) explants within 2 years of LVAD implant. Univariate analyses were done using Fine-Gray completing risks model and multivariable regression techniques including logistic regression, LASSO, RF, and GBM. The top 10 co-variates associated with time to all-cause mortality by p-value (Fine-Grey model, logistic regression) and strength of association (LASSO, RF, GBM) are shown in Table 2. Co-variates that were present in top 10 co-variates for at least 2 of the 5 methods were included in a final multivariable Fine-Grey model (Table 3, Central Illustration). The final model risk equation for waitlist mortality within 2 years post-LVAD implantation = 0.22*(age in years) + 0.11*BMI – 0.24*albumin + 0.06*total bilirubin + 0.1*natriuretic peptide + 0.21*BUN – 0.04*creatinine – 0.21*hemoglobin – 0.16*platelet + 0.1*WBC – 0.09*LVEDD + 0.09 (if right-sided heart failure present) – 0.03 (if neurologic dysfunction [any type] present) + 0.3 (if infection [all sources] present).

**Table 2:** Top 10 Candidate Predictors Associated with Time to All-cause Mortality Using Various Regression Techniques in the Derivation Cohort (INTERMACS).*

Rank*	Fine-Gray	Logistic Regression	LASSO	Random Forest	Gradient Boosting
1	Blood urea nitrogen	Right heart failure	Liver dysfunction	Blood urea nitrogen	Bilirubin
2	Creatinine	Age	Pump thrombosis	Bilirubin	Hemoglobin
3	Albumin	White blood cell count	Right heart failure	Creatinine	Platelets
4	Hemoglobin	LV end-diastolic dimension	Device malfunction	Hemoglobin	Blood urea nitrogen
5	Right heart failure	Hemoglobin	Neurologic dysfunction**	LV end-diastolic dimension	Creatinine
6	Natriuretic peptide	Body mass index	Supraventricular arrhythmia	Platelets	Albumin
7	White blood cell count	Infection – all sources	Blood type	Body mass index	Sodium
8	Major bleeding event	Neurologic dysfunction**	Primary diagnosis	Albumin	Cardiac index
9	Infection – all sources	Lactate dehydrogenase	Stroke	Natriuretic peptide	Body mass index
10	Age	Creatinine	Severe pulmonary disease	Age	Age

*Ranking of predictors based on p-value (Fine-Gray, logistic regression) or strength of association (LASSO, random forest, gradient boosting)

**Any type of neurologic dysfunction

**Table 3:** Final Risk Prediction Model for Waitlist Mortality in LVAD Patients Using a Multivariable Fine-Gray Model

Predictor	Estimate (SE)*	HR (95% CI)*
Age	0.22 (0.08)	1.25 (1.07, 1.47)
BMI	0.11 (0.07)	1.12 (0.97, 1.29)
Albumin (g/dL)	−0.24 (0.07)	0.78 (0.68, 0.9)
Total bilirubin (mg/dL)	0.06 (0.03)	1.06 (0.99, 1.14)
Natriuretic peptide	0.1 (0.07)	1.1 (0.97, 1.26)
Blood urea nitrogen (mg/dL)	0.21 (0.11)	1.23 (0.99, 1.53)
Creatinine (mg/dL)	−0.04 (0.11)	0.96 (0.77, 1.2)
Hemoglobin (g/dL)	−0.21 (0.07)	0.81 (0.71, 0.93)
Platelet (x10^3^/uL)	−0.16 (0.07)	0.85 (0.73, 0.98)
White blood cell (x10^3^/uL)	0.1 (0.05)	1.11 (1.01, 1.21)
LV end-diastolic diameter (cm)	−0.09 (0.07)	0.92 (0.8, 1.06)
Right heart failure	0.09 (0.17)	1.09 (0.78, 1.53)
Neurologic dysfunction (any type)	−0.03 (0.24)	0.97 (0.6, 1.56)
Infection (all sources)	0.3 (0.15)	1.35 (1.0, 1.83)

*Per 1-SD increase in continuous variables; if present (vs. absent) for categorical variables.

Risk of waitlist mortality in LVAD patients = 0.22*(age in years) + 0.11*BMI – 0.24*albumin + 0.06*total bilirubin + 0.1*natriuretic peptide + 0.21*BUN – 0.04*creatinine – 0.21*hemoglobin – 0.16*platelet + 0.1*WBC – 0.09*LVEDD + 0.09 (if right-sided heart failure present) - 0.03 (if neurologic dysfunction [any type] present) + 0.3 (if infection [all sources] present). BMI – body-mass index; BUN – blood urea nitrogen; LV – left ventricle; LVEDD – left ventricular end-diastolic diameter; WBC – white blood cell count.

### Model performance and validation

**Figure 2:**
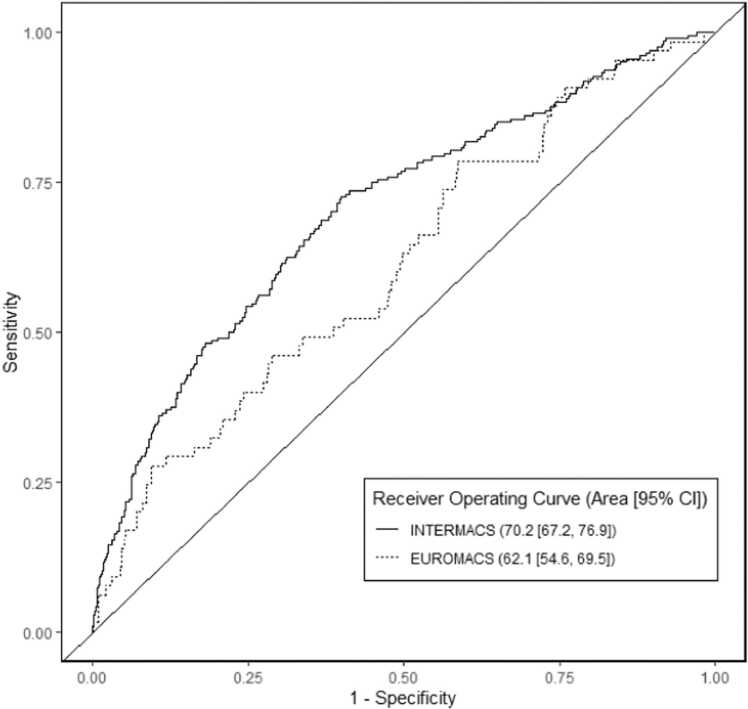
Receiver-operating characteristic curves for the risk assessment model predicting waitlist mortality in the INTERMACS and EUROMACS cohorts.

Receiver-operating characteristic (ROC) curves representing the ability of the model to assess time to all-cause mortality in INTERMACS and EUROMACS are shown in Figure 2. Model performance using INTERMACS had AUC 0.72 (95% CI 0.67–0.77).

In the EUROMACS cohort, there were 70 (12%) deaths, 142 (25%) transplants, and 7 (1%) explants within 2 years of LVAD implantation. Model performance using EUROMACS had AUC 0.62 (95% CI 0.55–0.70).

**Figure 3:**
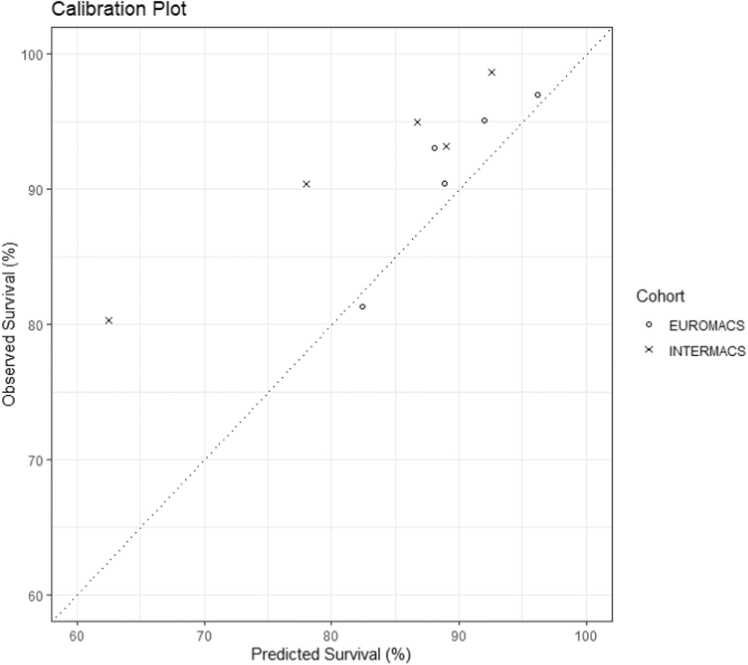
Calibration plot comparing predicted survival and observed survival at 2 years in the INTERMACS and EUROMACS cohorts. **Legend:** Markers represent quintiles of risk in each cohort. The Pearson correlation coefficient was 0.978 (p=0.004) in the INTERMACS cohort and 0.932 (p=0.02) in the EUROMACS cohort.

**Figure 4:**
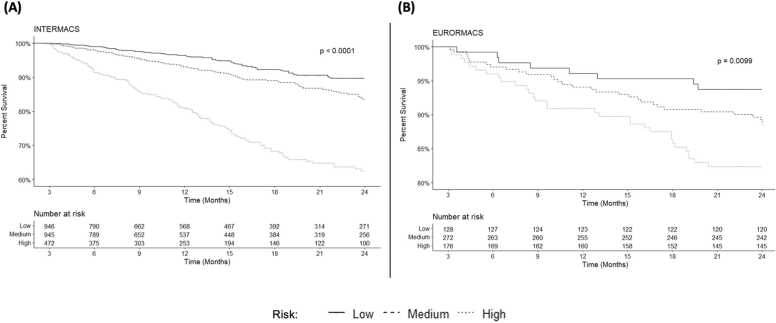
Observed patient survival at 2 year when stratified by risk profile for waitlist mortality. **Legend:** Kaplan-Meier survival of patients separated into low-, medium-, and high-risk for waitlist mortality using the risk assessment model in the (A) INTERMACS cohort and the (B) EUROMACS cohort.

To assess model calibration, predicted and observed survival was compared in the derivation and validation cohorts at 2 years (Figure 3). The Pearson coefficient was 0.978 with p=0.004 in INTERMACS and 0.932 with p=0.02 in EUROMACS. Patients were divided into groups based on low (0–3.4%), medium (3.4–7.2%), or high-risk (>7.2%) for waitlist mortality within 2 years. The 3 risk groups had a significant difference in observed waitlist mortality in the INTERMACS cohort (p<0.001) (Figure 4A) and the EUROMACS cohort (p=0.0099) (Figure 4B).

## Discussion

In this analysis, we developed and validated a risk assessment tool for waitlist mortality in LVAD patients being bridged to HT. There are several tools to evaluate risk of post-operative mortality following LVAD, largely designed to inform patient selection for LVAD implantation.^24^, ^25^, ^26^, ^27^ This study notably addresses risk assessment of mortality specifically in LVAD patients awaiting HT based on co-variates *following* LVAD implantation by utilizing real-world cohorts for model derivation and validation. Notably, the model was validated using the INTERMACS cohort and the entirely separate EUROMACS cohort.

The current Organ Procurement and Transplant Network (OPTN) criteria for HT categorizes LVAD patients in 4 discrete listing prioritizations. Those LVAD patients having experienced a device-related adverse event, requiring temporary mechanical support, or approved for a status upgrade exception are upgraded to relatively higher priority categories.^9^ Unfortunately, there is no further discrimination of patients based on risk of clinical deterioration within each category.^28^, ^29^, ^30^ A previously published analysis suggests that the current OPTN HT allocation scheme does not effectively stratify risk for waitlist mortality in LVAD patients.^14^ Several relevant clinical criteria that impact outcomes are not currently considered in the allocation scheme. Better discrimination of risk is necessary to allocate limited donor organs to LVAD patients most in need of them.

A significant advantage of our analysis was use of two completely unique large real-world cohorts of LVAD patients – the Interagency Registry for Mechanically Assisted Circulatory Support (INTERMACS) and European Registry for Patients with Mechanical Circulatory Support (EUROMACS).^15^, ^16^ These cohorts had comprehensive patient-level data allowing derivation and validation of the model to focus on patients with centrifugal-flow LVAD devices specifically listed or likely to be listed for HT. Although patient characteristics and practice patterns are different between the United States and Europe, having an external cohort for model validation is essential to avoid bias. Furthermore, our model leveraged multiple sophisticated statistical techniques for candidate predictor selection and utilized a completing risks time-to-event analysis best suited for risk prediction in a bridge-to-transplant population.^23^, ^31^, ^32^

Our risk assessment model for waitlist mortality in LVAD comprehensively incorporates several relevant clinical characteristics. The analysis started with 41 clinical co-variates of which 14 were selected in the final model. Although device-related adverse events are included in this model, many other significant risk factors for mortality were also incorporated.^10^, ^33^, ^34^, ^35^ This approach allows clinicians to identify and upgrade listing status for LVAD patients at higher risk of waitlist mortality that would otherwise remain at lower statuses in the current allocation scheme. Conversely, the model could also identify patients likely to have favorable outcomes while remaining on LVAD support and potentially in less need of a donor organ. Thus, a risk prediction model allows more precise discrimination of LVAD patients awaiting heart transplant based on risk of waitlist mortality. Our analysis also represents a step toward a heart allocation score in LVAD patients. Importantly, the model accurately categorizes patients by risk of waitlist mortality in the INTERMACS and EUROMACS cohorts.

Other risk assessment tools have been derived for LVAD patients. For example, the HeartMate 3 Risk Score (HM3RS) predicts survival up to 2 years following LVAD implantation based on pre-operative factors.^25^ This tool primarily informs patient selection for LVAD implantation itself. Many of the significant co-variates from the HM3RS (e.g. age, renal function, right heart failure) are present in our model as well. Another risk score using pre-operative risk factors and post-operative predictors from the index hospitalization was developed to predict long-term survival after LVAD implantation as bridge-to-transplant or destination therapy using a clinical trial cohort.^27^ However, our risk assessment model is designed specifically to evaluate risk of waitlist mortality in bridge-to-transplant LVAD patients. Moreover, these other risk scores were derived and validated using a single clinical trial cohort whereas our model is based on real-world cohorts including a completely unique cohort for external validation.

The association between relevant co-variates and mortality in LVAD patients can be attributed to several mechanisms. Notably, RHF in LVAD patients causes poor pump flow and increased venous congestion affecting other organs.^36^, ^37^, ^38^ In addition to RHF, increased BUN, increased bilirubin, decreased albumin, increased NP, and decreased LV end-diastolic dimension were also included in the model. These predictors are partly related to the severity of RHF and likely provide more insight into risk of waitlist mortality than solely the presence of RHF. Decreased albumin may be related to malnutrition, frailty, and a proinflammatory state.^39^, ^40^ Anemia, thrombocytopenia, and leukocytosis may be related to poor hemocompatibility and/or an inflammatory state.^41^, ^42^ Neurologic dysfunction including stroke and major infection are other predictors in this model that have also previously been associated with mortality in LVAD patients.^35^, ^43^, ^44^ In general, the characteristics in our final model are largely patient-specific rather than being related to the LVAD itself. Given this, the co-variates in our model likely remain relevant despite LVAD technology and management practices that have evolved since the study period.

There are several limitations in our risk assessment model. As discussed, there have been changes in LVAD technology and clinical practices since the study period. However, many of the co-variates are patient-specific and not directly related to pump characteristics. These co-variates likely remain relevant even as LVAD technology evolves particularly since many co-variates in our model are consistent with other risk models in patients awaiting HT with or without durable LVAD support.^25^, ^27^, ^45^ Although specific pump brand is not available in the registries, the study period would suggest a substantial cohort of patients with a Heartware HVAD™ were represented in the current analysis. Thus, future analyses with more HeartMate 3™ patients and subsequently with other LVAD devices that come to market will be necessary to iterate on this model.

Although the OPTN allocation scheme changed in 2018 during the study period, patient characteristics of LVAD patients listed for transplant have remained similar before and after the allocation change.^5^, ^8^, ^14^, ^46^ The factors used to increase prioritization amongst LVAD patients (e.g. LVAD-related adverse events and use of temporary mechanical circulatory support) remained consistent between the two allocation schemes.^9^ Even though further investigation is needed, these considerations suggest co-variates from our model remain relevant for LVAD patients in the current and potentially future allocation systems.

Even though INTERMACS and EUROMACS are enriched, comprehensive registries, inevitably certain relevant co-variates were not available for our analyses. For example, specific device brand (e.g. HeartMate 3™ or Heartware HVAD™) or use of temporary mechanical circulatory support while on durable LVAD support was not available. The accuracy of model may be limited by missing or inaccurate data in the registries.

Furthermore, discrepancies between INTERMACS and EUROMACS include variable definitions, data collection procedures, and geographic variability in clinical practice likely impacted model performance. In particular, our analysis showed significant differences between patients in the two registries pertaining to demographics, co-morbidities including renal dysfunction, degree of RV dysfunction and valvular disease, and LVAD-related adverse events including right heart failure, infection, neurologic dysfunction, and major bleeding. These differences likely contributed to the disparity in model performance between the two cohorts. As other registries start to contain more data about LVAD patients, future analyses can use better matched cohorts for external validation of risk models.

Further investigation will require large, prospective studies to improve model accuracy and performance. In particular, analyses with updated registry data will be essential for risk models to be relevant to contemporary practice. The HT allocation system has been an iterative process, and this analysis can inform subsequent versions of risk assessment models in LVAD patients particularly as the field moves toward a HT allocation score. Moreover, additional studies can determine how implementation of a risk assessment model for waitlist mortality impacts clinical outcomes including patient survival and organ utilization.

Overall, using a large, nationally representative cohort (INTERMACS) and a separate multicenter cohort (EUROMACS), we derived and validated a risk prediction for all-cause mortality within 2 years after LVAD implantation in patients on the waitlist for HT. The risk prediction model was based on a variety of complementary regression techniques including machine learning models. Although further prospective validation and iterative improvements of our model is necessary, our risk prediction model is an important step in informing donor heart allocation in LVAD patients awaiting HT.

## Declaration of Competing Interest

AT has received consulting fees from Boehringer Ingelheim and research funding from AstraZeneca, Omron Healthcare, and Cardiosense, Inc. JDR has received consulting fees from Boehringer Ingelheim and Lilly. FG has received consulting fees from Abbott, Alnylam, Amgen, AstraZeneca, Boehringer Ingelheim, Ionis, Novartis, Orion Pharma, and Pfizer. DTP has received consulting fees from Abbott, Medtronic, Abiomed. SJS has received research funding from AstraZeneca, Corvia, and Pfizer; and has received consulting fees from Abbott, Alleviant, Amgen, Aria CV, AstraZeneca, Axon Therapies, Bayer, Boehringer Ingelheim, Boston Scientific, BridgeBio, Bristol Myers Squibb, Corvia, Cytokinetics, Edwards Lifesciences, Eidos, Imara, Impulse Dynamics, Intellia, Ionis, Lilly, Merck, NGM Biopharmaceuticals, Novartis, Novo Nordisk, Pfizer, Prothena, Regeneron, Rivus, Sardocor, Shifamed, Tenax, Tenaya, and Ultromics. All other authors report no conflicts.

## Footnotes

Appendix A Supplemental data associated with this article can be found in the online version at doi:10.1016/j.jhlto.2025.100337.

## References

[1] Virani S.S., Alonso A., Aparicio H.J. (2021). Heart Disease and Stroke Statistics—2021 Update. Circulation.

[2] Feldman D., Pamboukian S.V., Teuteberg J.J. (2013). The 2013 International Society for Heart and Lung Transplantation Guidelines for mechanical circulatory support: executive summary. J Hear Lung Transpl.

[3] Mehra M.R., Canter C.E., Hannan M.M. (2016). The 2016 International Society for Heart Lung Transplantation listing criteria for heart transplantation: A 10-year update. J Hear Lung Transpl.

[4] Yuzefpolskaya M., Schroeder S.E., Houston B.A. (2022). The Society of Thoracic Surgeons Intermacs 2022 Annual Report: Focus on 2018 Heart Transplant Allocation System. Ann Thorac Surg.

[5] Hess N.R., Ziegler L.A., Keebler M.E., Hickey G.W., Kaczorowski D.J. (2023). Impact of 2018 Allocation System Change on Outcomes in Patients with Durable Left Ventricular Assist Device as Bridge to Transplantation: a UNOS Registry Analysis. J Heart Lung Transplant.

[6] Jorde U.P., Saeed O., Koehl D. (2024). THE SOCIETY OF THORACIC SURGEONS INTERMACS ANNUAL REPORT The Society of Thoracic Surgeons Intermacs 2023 Annual Report: Focus on Magnetically Levitated Devices The 14th Annual Report from The Society of Thoracic Surgeons (STS) Interagency Registry for Mec. Ann Thorac Surg.

[7] Zimpfer D., Gustafsson F., Potapov E. (2020). Two-year outcome after implantation of a full magnetically levitated left ventricular assist device: results from the ELEVATE Registry. Eur Heart J.

[8] Mullan C.W., Chouairi F., Sen S. (2021). Changes in use of left ventricular assist devices as bridge to transplantation with new heart allocation policy. JACC Hear Fail.

[9] Organ Procurement and Transplantation Network Data Reports. 2017. 〈https://optn.transplant.hrsa.gov/〉.

[10] Brouwers C., de Jonge N., Caliskan K. (2014). Predictors of changes in health status between and within patients 12 months post left ventricular assist device implantation. Eur J Hear Fail.

[11] Hasin T., Topilsky Y., Schirger J.A. (2012). Changes in renal function after implantation of continuous-flow left ventricular assist devices. J Am Coll Cardiol.

[12] Estep J.D., Starling R.C., Horstmanshof D.A. (2015). Risk assessment and comparative effectiveness of left ventricular assist device and medical management in ambulatory heart failure patients results from the ROADMAP Study. J Am Coll Cardiol.

[13] Tibrewala A., Wehbe R.M., Wu T. (2022). Hyponatremia is a powerful predictor of poor prognosis in left ventricular assist device patients. ASAIO J.

[14] Tibrewala A., Chuzi S., Wu T. (2024). Impact of heart transplant allocation change on waitlist mortality and posttransplant mortality in patients with left ventricular assist devices. Circ Hear Fail.

[15] Society of Thoracic Surgeons. Interagency Registry for Mechanically Assisted Circulatory Support. 〈http://www.uab.edu/medicine/intermacs〉.

[16] European Association for Cardiothoracic Surgery. European Registry for Patients with Mechanical Circulatory Support. 〈https://www.euromacs.org/home〉.

[17] Collins G.S., Reitsma J.B., Altman D.G., Moons K.G.M. (2015). Transparent reporting of a multivariable prediction model for individual prognosis or diagnosis (TRIPOD). Circulation.

[18] Yuzefpolskaya M., Schroeder S.E., Houston B.A. (2023). The Society of Thoracic Surgeons Intermacs 2022 Annual Report: Focus on the 2018 Heart Transplant Allocation System. Ann Thorac Surg.

[19] Malinchoc M., Kamath P.S., Gordon F.D., Peine C.J., Rank J., Ter Borg P.C.J. (2000). A model to predict poor survival in patients undergoing transjugular intrahepatic portosystemic shunts. Hepatology.

[20] Kamath P.S., Wiesner R.H., Malinchoc M. (2001). A model to predict survival in patients with end-stage liver disease. Hepatology.

[21] Kim W.R., Mannalithara A., Heimbach J.K. (2021). MELD 3.0: the model for end-stage liver disease updated for the modern era. Gastroenterology.

[22] White I.R., Royston P., Wood A.M. (2011). Multiple imputation using chained equations: Issues and guidance for practice. Stat Med.

[23] Fine J.P., Gray R.J. (1999). A proportional hazards model for the subdistribution of a competing risk. J Am Stat Assoc.

[24] Cowger J., Sundareswaran K., Rogers J.G. (2013). Predicting survival in patients receiving continuous flow left ventricular assist devices: the HeartMate II risk score. J Am Coll Cardiol.

[25] Mehra M.R., Nayak A., Morris A.A. (2022). Prediction of survival after implantation of a fully magnetically levitated left ventricular assist device. JACC Heart Fail.

[26] Loghmanpour N.A., Kanwar M.K., Druzdzel M.J., Benza R.L., Murali S., Antaki J.F. (2015). A new Bayesian network-based risk stratification model for prediction of short-term and long-term LVAD mortality. ASAIO J.

[27] Nayak A., Hall S.A., Uriel N. (2023). Predictors of 5-year mortality in patients managed with a magnetically levitated left ventricular assist device. J Am Coll Cardiol.

[28] Organ Procurement and Transplantation Network. Adult Heart Allocation. Accessed December 14, 2022. 〈https://optn.transplant.hrsa.gov/professionals/by-organ/heart-lung/adult-heart-allocation/〉.

[29] Silvestry S.C., Rogers J.G. (2022). Rinse, wash, repeat: The Evolution of the UNOS Heart Transplant Allocation System. JACC Heart Fail.

[30] Topkara V.K., Clerkin K.J., Fried J.A. (2021). Exception status listing in the new adult heart allocation system: a new solution to an old problem?. Circ Heart Fail.

[31] Austin P.C., Lee D.S., Fine J.P. (2016). Introduction to the analysis of survival data in the presence of competing risks. Circulation.

[32] Sapir-Pichhadze R., Pintilie M., Tinckam K.J. (2016). Survival analysis in the presence of competing risks: the example of waitlisted kidney transplant candidates. Am J Transplant.

[33] Kirklin J.K., Naftel D.C., Pagani F.D. (2015). Seventh INTERMACS annual report: 15,000 patients and counting. J Heart Lung Transpl.

[34] Teuteberg J.J., Cleveland J.C., Cowger J. (2020). THE SOCIETY OF THORACIC SURGEONS INTERMACS ANNUAL REPORT The Society of Thoracic Surgeons Intermacs 2019 Annual Report: the changing landscape of devices and indications. Ann Thorac Surg.

[35] Kirklin J.K., Pagani F.D., Kormos R.L. (2017). Eighth annual INTERMACS report: special focus on framing the impact of adverse events. J Hear Lung Transpl.

[36] Kormos R.L., Teuteberg J.J., Pagani F.D. (2010). Right ventricular failure in patients with the HeartMate II continuous-flow left ventricular assist device: Incidence, risk factors, and effect on outcomes. J Thorac Cardiovasc Surg.

[37] LaRue S.J., Raymer D.S., Pierce B.R., Nassif M.E., Sparrow C.T., Vader J.M. (2017). Clinical outcomes associated with INTERMACS-defined right heart failure after left ventricular assist device implantation. J Heart Lung Transplant.

[38] Shad R., Fong R., Quach N. (2021). Long-term survival in patients with post-LVAD right ventricular failure: multi-state modelling with competing outcomes of heart transplant. J Heart Lung Transpl.

[39] Imamura T., Combs P., Siddiqi U. (2020). Perioperative improvement in serum albumin level in patients with left ventricular assist device. J Card Surg.

[40] Muthiah K., Wilhelm K., Robson D. (2022). Impact of frailty on mortality and morbidity in bridge to transplant recipients of contemporary durable mechanical circulatory support. J Heart Lung Transplant.

[41] Shrout T., Sexton T., Vsevolozhskaya O., Guglin M., Shafii A., Smyth S. (2019). Early signatures of bleeding and mortality in patients on left ventricular assist device support: novel methods for personalized risk-stratification. Biomarkers.

[42] Vrtovec B., Radovancevic R., Delgado R.M. (2009). Significance of anaemia in patients with advanced heart failure receiving long-term mechanical circulatory support. Eur J Heart Fail.

[43] Willey J.Z., Gavalas M.V., Trinh P.N. (2016). Outcomes after stroke complicating left ventricular assist device. J Heart Lung Transpl.

[44] Healy A.H., Baird B.C., Drakos S.G., Stehlik J., Selzman C.H. (2013). Impact of ventricular assist device complications on posttransplant survival: an analysis of the United network of organ sharing database. Ann Thorac Surg.

[45] Zhang K.C., Narang N., Jasseron C. (2024). Development and validation of a risk score predicting death without transplant in adult heart transplant candidates. JAMA.

[46] Jani M., Lee S., Acharya D. (2022). Decreased frequency of transplantation and lower post-transplant survival free of re-transplantation in LVAD patients with the new heart transplant allocation system. Clin Transplant.

