# Supplementary material for: Risk prediction model for waitlist mortality in patients with left ventricular assist devices
Source: JHLT Open. 2025 Jul 19;10:100337. doi: 10.1016/j.jhlto.2025.100337 (PMC12362131; doi:10.1016/j.jhlto.2025.100337)
Supplement: Supplementary file 1 — Supplemental material [file mmc1.docx]

**SUPPLEMENTARY APPENDIX**

**INTERMACS Adverse Events Definitions**

Right heart failure:

﻿Definition: Symptoms or findings of persistent right ventricular failure characterized by both of the following:

● Documentation of elevated central venous pressure (CVP) by:

o Direct measurement (e.g., right heart catheterization) with evidence of a central venous pressure (CVP) or right atrial pressure (RAP) > 16 mmHg. or

o Findings of significantly dilated inferior vena cava with absence of inspiratory variation by echocardiography, or

o Clinical findings of elevated jugular venous distension at least half way up the neck in an upright patient.

● Manifestations of elevated central venous pressure characterized by:

o Clinical findings of peripheral edema (>2+ either new or unresolved), or

o Presence of ascites or palpable hepatomegaly on physical examination (unmistakable abdominal contour) or by diagnostic imaging, or

o Laboratory evidence of worsening hepatic (total bilirubin > 2.0 mg/dl) or renal dysfunction (creatinine > 2.0 mg/dl).

Device Malfunction:

﻿Definition: A Device Malfunction occurs when any component of the MCSD system ceases to operate to its designed performance specifications or otherwise fails to perform as intended.

Pump Thrombosis:

Definition: A special case of major device malfunction and can be delineated as suspected pump thrombus or confirmed pump thrombus. Pump thrombus will be classified as “SUSPECTED” based upon clinical, biochemical, or hemodynamic findings or “CONFIRMED” based upon device inspection or incontrovertible radiologic studies or absence of appropriate Doppler flow signals that confirms thrombus within the device or its conduits that results in or could potentially induce circulatory failure.

Infection- All Sources

Definition: ﻿A clinical infection accompanied by pain, fever, drainage and/or leukocytosis that is treated by anti-microbial agents (non-prophylactic). A positive culture from the infected site or organ should be present unless strong clinical evidence indicates the need for treatment despite negative cultures.

Infection- Pump/Driveline

Definition: Infection defined as one of the following categories.

- ﻿Percutaneous Site and/or Pocket Infection: A positive culture from the skin and/or tissue surrounding the drive line or from the tissue surrounding the external housing of a pump implanted within the body, coupled with the need to treat with antimicrobial therapy, when there is clinical evidence of infection such as pain, fever, drainage, or leukocytosis.
- Internal Pump Component, Inflow or Outflow Tract Infection: Infection of blood-contacting surfaces of the LVAD documented by positive site culture.

Neurologic Dysfunction

Definition: ﻿Any new, temporary or permanent, focal or global neurologic dysfunction ascertained by a standard neurological history and examination administered by a neurologist or other qualified physician and documented with appropriate diagnostic tests and consultation note; or an abnormality identified by surveillance neuroimaging. The examining physician will classify the event as a cerebrovascular event as defined below or as a non-vascular acute neurologic event. A neurologic event may be recognized by a clinically evident sign or symptom, or by clinically-silent electrographic seizure activity, or as a clinically silent lesion detected by surveillance neuroimaging.

Neurologic Dysfunction- Stroke

Definition: Stroke defined as any one of the following categories.

﻿

- Ischemic stroke, defined as a new acute neurologic deficit (or acute encephalopathy or seizures in children <6 months**) of any duration associated with acute infarction on imaging corresponding anatomically to the clinical deficit. Ischemic stroke should be sub classified as due to arterial-distribution ischemia or due to venous thrombosis.
- Acute symptomatic intracranial hemorrhage, defined as new acute neurologic deficit attributable to Intracranial hemorrhage (ICH).
- Clinically covert ischemic stroke or ICH: infarction or ICH seen by surveillance imaging, without clinical findings of stroke or ICH at the time of event recognition.

Major Bleeding Event:

Definition: ﻿An episode of SUSPECTED INTERNAL OR EXTERNAL BLEEDING that results in one or more of the following: Death, Re-operation, Hospitalization, or Transfusion of red blood cells.

**EUROMACS Adverse Events Definition**

Right Heart Failure

Definition: Symptoms or signs of right heart failure occur requiring RVAD implantation or inotropic therapy at least 14 days post implant and since last update.

Device Malfunction

Definition: A Device Malfunction occurs when any component of the VAD system does not function properly, is positioned, and/or requires replacement.

Pump Thrombosis

Definition: A particular case of major device malfunction and can be delineated as device thrombus.

Infection – All Sources

Definition: Clinical evidence of infection categorized as pump related, positive blood cultures, line sepsis, pulmonary, urinary tract infection, mediastinum, peripheral wound, GI, or other.

Infection – Pump/Driveline

Definition: Clinical evidence of infection related to drive line, pump pocket, and/or pump interior.

Neurologic Dysfunction

Definition: Clinical evidence of neurologic dysfunction categorized as neurologic dysfunction <24 hours, neurologic dysfunction >24 hours, abnormality of head ultrasound, positive EEG, other neurologic dysfunction.

Neurologic Dysfunction – Stroke

Definition: Clinical evidence of neurologic dysfunction with objective evidence of stroke.

Major Bleeding Event:

Definition: ﻿An episode of bleeding that results in one or more of the following: Death, Re-operation, Re-admission, or Transfusion of red blood cells.
